# Ultrasound-guided evaluation of the lumbar subarachnoid space in lateral and sitting positions in pregnant patients to receive elective cesarean operation

**DOI:** 10.12669/pjms.311.5647

**Published:** 2015

**Authors:** Ucarli Gulay, Turkay Meltem, Sinikoglu Sitki Nadir, Alagol Aysin

**Affiliations:** 1 Ucarli Gulay, Medical Doctor, Department of Anesthesiology and Reanimation, Ardahan State Hospital, Turkey.; 2 Turkay Meltem, Medical Doctor, Bagcilar Training and Research Hospital.; 3 Sinikoglu Sitki Nadir, Medical Doctor, Bagcilar Training and Research Hospital.; 4 Alagol Aysin, Associate Professor,

**Keywords:** Cesarean Section, Spinal Anesthesia, Ultrasound

## Abstract

**Background and Objective:**

The aim was to compare visibility of the spinal space in sitting and lateral positions, number of attempts, spinal needle depth, skin-dura mater distance and the possible complications; in application of spinal anesthesia, using ultrasound in pregnant patients scheduled to receive elective cesarean operations.

**Methods:**

The study was conducted prospective-randomly after receiving approval from the ethics committee and the patients’ permission. ASA I-II 50 pregnant patients were divided into two groups. The patients in Group SP were those placed in a sitting position and the patients in Group LP were those placed in a lateral position. In both groups, the skin-dura mater distance was recorded through an out-of plane technique accompanied by ultrasound. The depth of the spinal needle was measured. The number of attempts, the level of attempts recorded. The degree of visibility of the vertebral space was observed through ultrasound and was numerically scored. Intraoperative and postoperative complications were recorded.

**Results:**

There was no difference between the number of attempts, Modified Bromage Scale and mean measurements of skin-dura mater distance observed through ultrasound. The mean needle depths of Group LP were statistically found significantly higher than Group SP (p=0.002).

**Conclusion:**

Our study supports the notion that access to the skin-dura mater distance is longer in the lateral decubitus position when skin-dura mater distance is evaluated by measuring needle depth.

## INTRODUCTION

Maternal mortality and morbidity were significantly reduced by using neuroaxial blocks in obstetric anesthesia.^1^ Spinal anesthesia is a frequently used technique since it creates a quick deep sensory and motor block through the injection of a low dose of local anesthetic to the subarachnoid space.^2^ In recent years, it has become known that the use of ultrasound in regional anesthesia increases block success and decreases complications.^3^ Ultrasound enables accurate estimation of the depth required to reach the intrathecal space.^4^

The primary objective of our study was to compare the visibility of spinal space, number of attempts, spinal needle length and skin-dura mater distance measured in sitting and lateral positions during spinal anesthesia applied with the use of ultrasound, to pregnant patients about to receive elective cesarean operation; and our secondary objective was to determine the effect of the lateral and sitting positions on the frequency of possible complications.

## METHODS

The study was prospective and randomly conducted on 50 pregnant patients who did not have pregnancy complications, were aged 18 or over, are of ASA I-II group, had a gestation age over 37 weeks and would receive elective cesarean operation under spinal anesthesia, after obtaining ethics committee approval and written permissions from patients. Elective cases with 8 hours of fasting were included in the study. Emergency cases, patients with spinal anesthesia contraindication (coagulopathy, infection in application site etc.) and patients who did not want spinal anesthesia were excluded from the study.

A peripheric vascular access was opened in all patients in the preoperative patient room before the attempt from the dorsum of the hand or antecubital area with an 18 gauge intravenous (iv) cannula; and 500-750 ml liquid calculation was made with 0.9% NaCI infusion. The patients taken to the operating table received standard monitoring. Three-channel ECG, blood pressure through noninvasive method (systolic, diastolic, mean artery pressures), heart pulse rate and peripheric oxygen saturation (SpO_2_) values were tracked. During monitoring, the patients were taken into 15-20^0^ left lateral position in order to prevent aorta hollow pressure.

Before the block application, demographic data (age, height, weight before pregnancy, last body weight, gestation time, number of pregnancy, and number of births) of all of the pregnant patients were recorded. General anesthesia conditions and 0.50 mg atropine sulfate and 10 mg ephedrine were made available to all patients before application.

The patients were randomized into the groups with the computer by an anaesthesia nurse. Patients receiving spinal anesthesia with ultrasound in sitting position were named as Group SP, and the patients receiving spinal anesthesia with ultrasound in lateral position were named as Group LP. All the attempts were performed by a single doctor. Two assistants were utilized during the application.

Patients in Group SP were placed into the required sitting position after they sat on the edge of the operating table by suspending their feet and stepping on a stool. The patients in Group LP were put into the lateral position and pillows were placed under their heads and shoulders. The attempt site and the ultrasound probe were prepared in a sterile manner for the patients in both groups. Lumbar vertebral distances were palpated using the anatomic indicators by anesthesiologist experienced for more than 5 years (an imaginary line passing from spina iliaca posterior superior was accepted to pass through L4-L5 distance). Vertebral spaces were confirmed via spinous processes. The patients were asked to flex their heads and lean their heads to their chests and knit their arms in front of their bodies; and their legs became flex and lower backs became flat.

**Fig.1:**
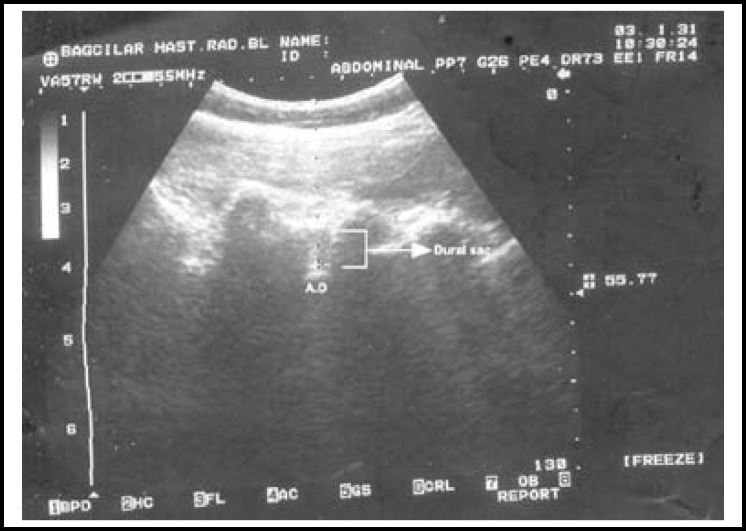
Ultrasonic image of sonoanatomic structures in intervertebral space and skin-dura mater distance.

**Fig.2:**
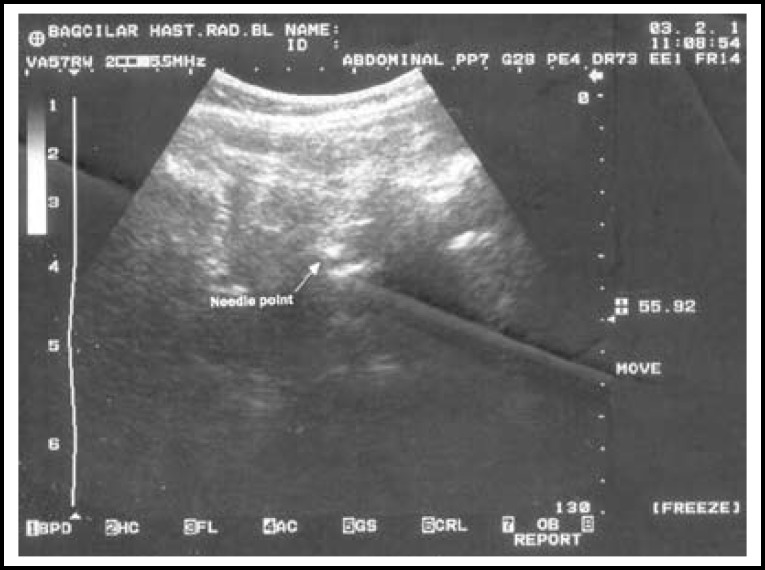
Ultrasonic image of the needle point

Lumbar ultrasound was applied using 2-5.5 MHz convex probe with an ultrasound device (Shimadzu, SDU-450 XL, Kyoto, JAPAN). The probe was first placed in the sacral region at 2-3 cm away from the middle line and paramedian longitudinal. The sacrum was observed as a ceaseless hyperecoic line. The probe was routed against the cranial in order to see the vertebral processes. Since the spinous processes of lumbar vertebras look like the teeth of a saw, intervertebral distances were observed hypoechoic. The sonoanatomic structures in intervertebral space were detected through ultrasound (Fig.1). The short ax (out-of plane) needle placement technique was used with ultrasound. The intrathecal space was entered through passing the skin, sub-skin and dura mater with median approach and 25 G Quincke needle (Exelint/California/USA) from the lumbar space. It was observed through ultrasound that the needle pierced the dura mater and reached the subarachnoid distance. The point of the needle was observed as a shiny point on the ultrasound (Fig.2). The measurement of skin-dura mater distance was recorded. After the clear cerebrospinal fluid (CSF) flow was detected, spinal anesthesia was applied with 10 mg hyperbaric bupivacaine (Marcaine heavy 0.5%, Astra Zeneca, Turkey). While the spinal needle was drawn back, it was kept firmly at the skin level and marked with a sterile skin-marker pen. The measurement of skin-dura mater distance was recorded as needle depth in cm. During application, one of the assistants waited in front of the patient and provided assistance to ensure the patient maintained the position.

A subarachnoid block was applied from the L3-L4 or L4-L5 space where lumbar vertebral space palpation is the best and the ultrasound image is observed most clearly. In cases where the attempt was not possible, the block was applied from the L2-L3 space.

All patients were taken into left-tilt and supine position after the application. Pillows were placed under their heads and shoulders. If the blood pressure of the patient recorded a decrease by more than 25% than that of the beginning value, or the mean blood pressure was below 90 mmHg, hypotension was accepted to be present and recorded. Fast crystalloid liquid infusion and repeated doses of iv 5 mg ephedrine were administered to patients developing hypotension. Decrease in the number of heart pulses below 50 pulse min^-1^ was accepted as bradycardia and was recorded. 0.5 mg atropine was administered to patients developing bradycardia. The number of attempts for each patient, the space from which the attempt was applied, whether static click was felt during attempt and whether there was clear CSF flow were recorded. Visibility degrees of the anatomic structures in vertebral space (spinous process, vertebral bone, ligamentum flavum, dura mater, static bladder) observed through ultrasound, were numerically scored using the following values:

0: Anatomic structures cannot be observed at all.

1: Anatomic structures can be slightly observed.

2: Anatomic structures can be observed well.

3: Anatomic structures can be observed very well.

Whether the lumbar vertebral space receiving the attempt could be felt through palpation was determined as good/bad/medium and was recorded. Whether any intraoperative (nausea, vomiting, hypotension, bradycardia) and postoperative (headache, neurological complication etc.) complications developed was recorded.

Sensorial block levels of patients were evaluated with the “pin-pick” test applied in 5 min intervals. A Modified Bromage Scale^5^ was used to evaluate the motor block.

The patients were taken to the recovery unit after the operation. The patients were then sent to Department of Obstetrics and Gynaecology after hemodynamic findings became stable, motor block was totally removed (Bromage 0) and the sensory block decreased to T10 level.

All patients were questioned one day after the surgical operation in relation to issues with head ache, motor and neurological deficit.

***Statistical Evaluation: ***In evaluation of the data, in addition to definitive statistical methods (median, standard deviation), independent t-test was used in the comparison of dual groups and chi-square test was used in the comparison of qualitative data. The results were evaluated at p<0.05 significance level.

## RESULTS

There was no statistically significant difference between the groups’ age, height means and ASA distributions and the groups’ body weight before pregnancy, current body weight, number of pregnancies, number of live births and pregnancy period means. (p>0.05) (Table-I).

Similarly no statistically significant difference was observed between the groups’ number of attempts, ultrasonic measurement of skin-dura mater distance and Modified Bromage Scale means (p>0.05). The needle depth means in Group LP were significantly higher as compared to Group SP in statistical terms (p=0.002) (Table-II).

There was no statistically significant difference between comorbid diseases, intraoperative and postoperative complication distributions of the groups (p>0.05) (Table-III).

There was no statistically significant difference between spinal anesthesia attempt level and unsuccessful block distributions of the groups (p>0.05) (Table-IV) and no statistically significant difference between groups in terms of visibility of anatomic structures in vertebral space through ultrasound and palpation of the vertebral space (p>0.05) (Table-V).

In addition no statistically significant difference was noted between groups in terms of distribution of block levels and developed intraoperative complications (p>0.05) (Table-VI).

## DISCUSSION

In recent years, ultrasound has been presented as an innovative and promising device to facilitate neuroaxial anesthesia application and it is stated that significant information can be obtained pertaining to spinal anatomy through the use of ultrasound.^6^ Ultrasound is proposed to be used in preoperative evaluations particularly in patients expected to demonstrate technical difficulties in neuroaxial blocks.^7^

In certain studies, it is stated that palpation is traditionally used in detecting the lumbar vertebral space^8^, but the level could not be detected accurately through palpation and this could increase complications such as neurological damage and paralysis.^9^ In one study, the intervertebral space determined by ultrasound and palpation was marked with ultraviolet indicators and examined in x–ray. As a result of this study, while ultrasound imaging demonstrated the accurate level in 71% of the patients, palpation demonstrated only 30% success.^10^ In another study, the accuracy rate of determining intervertebral space through ultrasound was reported to be 76%.^11^ Whitty et al.^12^ evaluated postpartum, patients receiving obstetric neuroaxial anesthesia through palpation. In these patients, it was detected that the level determined by palpation was actually one to two levels above that seen when observed with ultrasound. Schlotterbeck et al.^13^ evaluated pregnant patients receiving lumbar neuroaxial anesthesia with ultrasound after determining the attempt level in accordance with needle puncture sites. They detected that the clinical accuracy was 36.4%, upper level of attempt when the stated was applied in more than 50% of the patients and lower level of attempt was applied in 15% of them. They drew attention to the fact that attempts over the L3 level are more risky in terms of neurological complications and to these important risks that may develop together with the increase in techniques comprising static puncture in anesthesia of pregnant patients^13^. Locks et al.^14^, on the contrary, did not detect any difference between level detections through palpation and ultrasound. In our study, the location to apply the attempt at the lumbar vertebral level was determined through ultrasound. Rather than detection of vertebral levels through palpation, palpability of lumbar vertebral spaces on skin was sought. There was no significant difference between groups in terms of visibility degree through ultrasound with position, palpation and block levels.

In certain studies, the effects of sitting and lateral position on hemodynamics and block in pregnant patients receiving regional anesthesia were researched.^15^^,^^16^ In their study, Khurrum et al.^15^ examined 70 patients aged below 60 that would receive spinal anesthesia. They found similar effects in sitting and lateral positions in terms of sensory, motor block and hemodynamic stability; but detected that the lateral position was more comfortable for patients.^15^ Inglis et al.^17^ reported that spinal anesthesia is more quickly applied in a sitting position and less ephedrine is needed within the first 10 minutes after spinal injection. In our study, there was no significant difference between intraoperative and postoperative complications resulting from position in spinal anesthesia application performed in accompany with ultrasound. Furthermore, there was no significant difference between block levels. Although one patient from Group SP demonstrated good imaging through ultrasound, felt the static click and demonstrated a clear CSF flow, the spinal block was unsuccessful. The patient subsequently received general anesthesia.

It has been reported that ultrasound is the golden standard in determining the epidural space and being aware of the skin-epidural distance and skin-subarachnoid distance helps to decrease the risk of accidental static piercing during the process.^18^ Palmer et al.^19^ reported in their study that skin-epidural distance measurement, in epidural block application in obstetric patients, is directly related to body weight and the changes in the tissue under the skin are the most important factor in measurement of the skin-epidural distance. Gnaho et al.^4^ applied spinal anesthesia in sitting position at lumbar L3-L4 level and found skin-anterior ligamentum flavum distance and spinal needle depth as (5.154±0.95 cm) and (5.14±0.97 cm) respectively.^4^

Bassiakou et al.^20^ measured skin-epidural distance, skin-subarachnoid distance and epidural-subarachnoid distance in combined spinal epidural anesthesia application in left lateral position at the L3-L4 space. They determined the distances as (5.6±1.6 cm), (6.5±1.2 cm) and (0.9±0.5 cm) respectively and reported that the correlation between these physical and anthropometric measurements could have a potential value for pregnant patients.^20^ Hamza et al.^21^ evaluated the skin-epidural distance in sitting and left lateral positions with needle depth. They detected that there was a positive correlation between height and body mass index and skin-epidural distance and the skin-epidural distance depth increased significantly (approximately 0.5 cm) in left lateral position as compared to sitting position. The skin-epidural distance measurements in sitting and lateral position were found to be (4.44±0.82 cm) and (5.03±1.05 cm) respectively.^21^ In our study, the skin-spinal space distances detected with ultrasound in Group SP and Group LP were (5.47±0.56 cm) and (5.65±0.51 cm) respectively and the needle depth measurements were (5.52±0.69 cm) and (6.25±0.92 cm) respectively. The needle depth was found to be significantly longer in Group LP. As also reported by Bassiakou et al.^20^, although there are many studies on skin-epidural distance in obstetric patients, the number of studies searching for skin-subarachnoid distance is quite limited. We are of the opinion that other studies are required to evaluate the reasons for the differences in skin-subarachnoid distance and needle depth measurements depending on the position.

AD: Anterior dura mater – ligamentum flavum complex.

**Table-I:** Age, height, ASA distributions, body weight before pregnancy, current body weight, number of pregnancies, number of live births and pregnancy period in Group SP and Group LP

		**Group SP** **(n=25)**		**Group LP** **(n=25)**		**p**
Age (year)		29.76±5.91		30.84±6.07		0.527
Height (cm)		159.64±5.98		161.76±6.19		0.224
ASA	I	23	92.00%	22	88.00%	0.637
	II	2	8.00%	3	12.00%	
Body weight before pregnancy (kg)		66.88±10.74		67.2±12. 9		0.924
Current body weight (kg)		77.12±11.34		76.56±11.53		0.863
Number of pregnancies (n)		2.76±1.27		2.88±1.13		0.725
Number of live births (n)		1.52±1.01		1.54±0.76		0.998
Pregnancy period (day)		276.64±4.63		276.48±3.54		0.891

Mean±SD, ASA: American Society of Anesthesiologists.

**Table-II:** **N**umber of attempts, ultrasonic measurement of skin-dura mater distance, needle depth and Modified Bromage Scale means in Group SP and Group LP.

	**Group SP** ** (n=25)**	**Group LP** ** (n=25)**	**p**
Number of attempts	2.16±0.85	2.08±0.7	0.718
Ultrasonic measurement of skin-dura mater distance (cm)	5.47±0.56	5.65±0.51	0.241
Needle depth (cm)	5.52±0.69	6.25±0.92	0.002*
Modified Bromage Scale	2.56±0.77	2.88±0.33	0.062

p<0.05 (Mean±SD)

**Table-III:** Comorbid diseases, intraoperative and postoperative complication distributions of the groups

		**Group SP** **(n=25)**		**Group LP** **(n=25)**		**p**
Comorbid diseases	Absent	18	72.00%	18	72.00%	0.100
	Present	7	28.00%	7	28.00%	
Intraoperative Complication	Absent	13	52.00%	9	36.00%	0.393
	Present	12	48.00%	16	64.00%	
Postoperative Complication	Absent	25	100.00%	23	92.00%	0.149
	Present	0	0.00%	2	8.00%	

**Table-IV:** Vertebral level of dural puncture and unsuccessful block distributions of the groups

		**Group SP** **(n=25)**		**Group LP** **(n=25)**		**P**
Attempt level	L4-L5	15	60.00%	18	72.00%	0.195
	L3-L4	7	28.00%	7	28.00%	
	L2-L3	3	12.00%	0	0.00%	
Unsuccessful block	Absent	24	96.00%	25	100.00%	0.312
	Present	1	4.00%	0	0.00%	

**Table-V:** Groups in terms of visibility of anatomic structures in vertebral space through ultrasound and palpation of the vertebral space

		**Group SP** **(n=25)**		**Group LP** **(n=25)**		**p**
Visibility of anatomic structures in vertebral space through ultrasound	Slightly	5	20.00%	6	24.00%	0.850
	Well	15	60.00%	13	52.00%	
	Very well	5	20.00%	6	24.00%	
Palpation of the vertebral space	Bad	3	12.00%	5	20.00%	0.632
	Medium	8	32.00%	9	36.00%	
	Good	14	56.00%	11	44.00%	

**Table-VI:** Groups in terms of distribution of block levels and developed intraoperative complications

		**Group SP** **(n=25)**		**Group LP** **(n=25)**	
Block Level	Unsuccessful block	1	4.00%	0	0.00%
	T2	0	0.00%	2	8.00%
	T4	8	32.00%	16	64.00%
	T5	1	4.00%	0	0.00%
	T6	4	16.00%	3	12.00%
	T7	1	4.00%	4	16.00%
	T8	10	40.00%	0	0.00%
Intraoperative Complication	No complication	13	52.00%	9	36.00%
	Nausea	5	20.00%	3	12.00%
	Hypotension	2	8.00%	5	20.00%
	Nausea + Hypotension	4	16.00%	6	24.00%
	Nausea+Vomiting+Hypotension	0	0.00%	1	4.00%
	Hypotension+Bradycardia	1	4.00%	1	4.00%

Schnabel et al.^22^ detected that ultrasound provides less number of attempts. They stated that the success rate in the first attempt was 71% more as compared to the resistance loss technique and this reduced the complication rate. Grau et al.^23^ reported that the complication rate in pregnant patients receiving epidural anesthesia and analgesia is 20% and stated that the use of ultrasound decreased the number of attempts as compared to the control group. In our study, there was no significant difference between the two groups in terms of attempt level distributions and the number of attempts. Again, there is no statistically significant difference between the groups in terms of visibility of the structures in the vertebral space through ultrasound.

## CONCLUSION

Increased body weight and subcutaneous sub-skin tissue edema can affect the measurement of skin-dura mater distance. Change of the epidural depth with position is an important factor in the measurement of skin-dura mater distance. We did not detect any significant difference between skin-dura mater distance measurements in lateral decubitus and sitting positions. In evaluation of the skin-dura mater distance with needle depth measurements, our study supports the claim that access to the skin-dura mater distance is longer in lateral decubitus position.

## Authors’ Contribution:

**GU** conceived, designed, data collection, and manuscript writing.

**MT **did data collection and manuscript writing.

**SNS** did editing of manuscript.

**AA** did review and final approval of manuscript.
